# CIVIL MONETARY PENALTIES GOVERNING ASSISTED LIVING: VARIATION ACROSS AND WITHIN STATES

**DOI:** 10.1093/geroni/igad104.0211

**Published:** 2023-12-21

**Authors:** Jacy Weems, Lindsey Smith, Cassandra Hua, Kali Thomas

**Affiliations:** Brown University School of Public Health, Providence, Rhode Island, United States; Oregon Health & Science University, Portland, Oregon, United States; University of Massachusetts Lowell, Lowell, Massachusetts, United States; Johns Hopkins University, Baltimore, Maryland, United States

## Abstract

State survey agencies are responsible for developing minimum requirements for assisted living (AL) communities, including penalties for noncompliance. Civil monetary penalties are a common measure state enforcement agencies use to incentivize regulatory compliance. We searched state statutes and regulations governing licensed AL communities in August 2022 to identify enforceable civil monetary penalties and used descriptive statistics and content analysis to describe regulatory differences within and between states according to license type. Our findings indicate that 48 of 51 states may enforce financial penalties for noncompliance against assisted living communities, though four of these states have one or more license types without applicable monetary penalties. 13 states had differing policies and possible enforceable amounts by license type, so some AL communities are possibly being charged more than others for the same offense within the same state. We found that states took three different approaches to calculating fees, sometimes using multiple approaches depending on the citation and license type. One or more license types in 36 states imposed per-instance fines, per-day fines in 32 states, and per-resident-per-day fines in 3 states. We describe the current landscape of financial penalty enforcement policies for US assisted living communities as well as its implications for state agencies and providers.

